# Defining Feasibility and Pilot Studies in Preparation for Randomised Controlled Trials: Development of a Conceptual Framework

**DOI:** 10.1371/journal.pone.0150205

**Published:** 2016-03-15

**Authors:** Sandra M. Eldridge, Gillian A. Lancaster, Michael J. Campbell, Lehana Thabane, Sally Hopewell, Claire L. Coleman, Christine M. Bond

**Affiliations:** 1 Centre for Primary Care and Public Health, Queen Mary University of London, London, United Kingdom; 2 Department of Mathematics and Statistics, Lancaster University, Lancaster, Lancashire, United Kingdom; 3 School of Health and Related Research, University of Sheffield, Sheffield, South Yorkshire, United Kingdom; 4 Clinical Epidemiology and Biostatistics, McMaster University, Hamilton, Ontario, Canada; 5 Nuffield Department of Orthopaedics, Rheumatology and Musculoskeletal Sciences, University of Oxford, Oxford, Oxfordshire, United Kingdom; 6 Centre of Academic Primary Care, University of Aberdeen, Aberdeen, Scotland, United Kingdom; Azienda Ospedaliero-Universitaria Careggi, ITALY

## Abstract

We describe a framework for defining pilot and feasibility studies focusing on studies conducted in preparation for a randomised controlled trial. To develop the framework, we undertook a Delphi survey; ran an open meeting at a trial methodology conference; conducted a review of definitions outside the health research context; consulted experts at an international consensus meeting; and reviewed 27 empirical pilot or feasibility studies. We initially adopted mutually exclusive definitions of pilot and feasibility studies. However, some Delphi survey respondents and the majority of open meeting attendees disagreed with the idea of mutually exclusive definitions. Their viewpoint was supported by definitions outside the health research context, the use of the terms ‘pilot’ and ‘feasibility’ in the literature, and participants at the international consensus meeting. In our framework, pilot studies are a subset of feasibility studies, rather than the two being mutually exclusive. A feasibility study asks whether something can be done, should we proceed with it, and if so, how. A pilot study asks the same questions but also has a specific design feature: in a pilot study a future study, or part of a future study, is conducted on a smaller scale. We suggest that to facilitate their identification, these studies should be clearly identified using the terms ‘feasibility’ or ‘pilot’ as appropriate. This should include feasibility studies that are largely qualitative; we found these difficult to identify in electronic searches because researchers rarely used the term ‘feasibility’ in the title or abstract of such studies. Investigators should also report appropriate objectives and methods related to feasibility; and give clear confirmation that their study is in preparation for a future randomised controlled trial designed to assess the effect of an intervention.

## Introduction

There is a large and growing number of studies in the literature that authors describe as feasibility or pilot studies. In this paper we focus on feasibility and pilot studies conducted in preparation for a future definitive randomised controlled trial (RCT) that aims to assess the effect of an intervention. We are primarily concerned with stand-alone studies that are completed before the start of such a definitive RCT, and do not specifically cover internal pilot studies which are designed as the early stage of a definitive RCT; work on the conduct of internal pilot studies is currently being carried out by the UK MRC Network of Hubs for Trials Methodology Research. One motivating factor for the work reported in this paper was the inconsistent use of terms. For example, in the context of RCTs ‘pilot study’ is sometimes used to refer to a study addressing feasibility in preparation for a larger RCT, but at other times it is used to refer to a small scale, often opportunistic, RCT which assesses efficacy or effectiveness.

**Fig 1:**
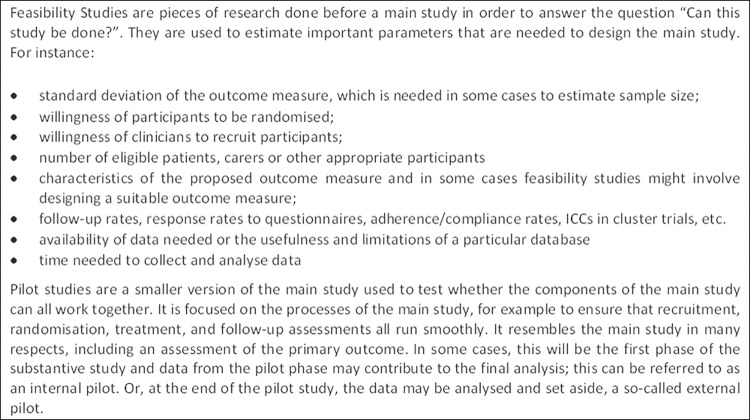
NIHR definitions [5, 6].

A second, related, motivating factor was the lack of agreement in the research community about the use of the terms ‘pilot’ and ‘feasibility’ in relation to studies conducted in preparation for a future definitive RCT. In a seminal paper in 2004 reviewing the literature in relation to pilot and feasibility studies conducted in preparation for an RCT [1], Lancaster *et al* reported that they could find no formal guidance as to what constituted a pilot study. In the updated UK Medical Research Council (MRC) guidance on designing and evaluating complex interventions published four years later, feasibility and pilot studies are explicitly recommended, particularly in relation to identifying problems that might occur in an ensuing RCT of a complex intervention [2]. However, while the guidance suggests possible aims of such studies, for example, testing procedures for their acceptability, estimating the likely rates of recruitment and retention of subjects, and the calculation of appropriate sample sizes, no explicit definitions of a ‘pilot study’ or ‘feasibility study’ are provided. In 2010, Thabane and colleagues presented a number of definitions of pilot studies taken from various health related websites [3]. While these definitions vary, most have in common the idea of conducting a study in advance of a larger, more comprehensive, investigation. Thabane *et al* also considered the relationship between pilot and feasibility, suggesting that feasibility should be the main emphasis of a pilot study and that ‘a pilot study is synonymous with a feasibility study intended to guide the planning of a large scale investigation’. However, at about the same time, the UK National Institute for Health Research (NIHR) developed definitions of pilot and feasibility studies that are mutually exclusive, suggesting that feasibility studies occurred slightly earlier in the research process and that pilot studies are ‘a version of the main study that is run in miniature to test whether the components of the main study can all work together’. Arain *et al*. felt that the NIHR definitions were helpful, and showed that studies identified using the keyword ‘feasibility’ had different characteristics from those identified as ‘pilot’ studies [4]. The NIHR wording for pilot studies has been changed more recently to ‘a smaller version of the main study used to test whether the components of the main study can all work together’ (Fig 1). Nevertheless, it still contrasts with the MRC framework guidance that explicitly states: ‘A pilot study need not be a “scale model” of the planned main-stage evaluation, but should address the main uncertainties that have been identified in the development work’ [2]. These various, sometimes conflicting, approaches to the interpretation of the terms ‘pilot’ and ‘feasibility’ exemplify differences in current usage and opinion in the research community.

While lack of agreement about definitions may not necessarily affect research quality, it can become problematic when trying to develop guidance for research conduct because of the need for clarity over what the guidance applies to and therefore what it should contain. Previous research has identified weaknesses in the reporting and conduct of pilot and feasibility studies [1, 3, 4, 7], particularly in relation to studies conducted in preparation for a future definitive RCT assessing the effect of an intervention or therapy. While undertaking research to develop guidance to address some of the weaknesses in reporting these studies, we became convinced by the current interest in this area, the lack of clarity, and the differences of opinion in the research community, that a re-evaluation of the definitions of pilot and feasibility studies was needed. This paper describes the process and results of this re-evaluation and suggests a conceptual framework within which researchers can operate when designing and reporting pilot/feasibility studies. Since our work on reporting guidelines focused specifically on pilot and feasibility studies in preparation for an RCT assessing the effect of some intervention or therapy, we restrict our re-evaluation to these types of pilot and feasibility studies.

## Methods

The process of developing and validating the conceptual framework for defining pilot and feasibility studies was, to a large extent, integral to the development of our reporting guidelines, the core components of which were a large Delphi study and an international expert consensus meeting focused on developing an extension of the 2010 CONSORT statement for RCTs [8] to randomised pilot studies. The reporting guidelines, Delphi study and consensus meeting are therefore referred to in this paper. However, the reporting guidelines will be reported separately; this paper focuses on our conceptual framework.

### Developing a conceptual framework—Delphi study

Following research team discussion of our previous experience with, and research on, pilot and feasibility studies we initially produced mutually exclusive definitions of pilot and feasibility studies based on, but not identical to, the definitions used by the NIHR. We drew up two draft reporting checklists based on the 2010 CONSORT statement [8], one for what we had defined as feasibility studies and one for what we had defined as pilot studies. We constructed a Delphi survey, administered on-line by Clinvivo [9], to obtain consensus on checklist items for inclusion in a reporting guideline, and views on the definitions. Following user-testing of a draft version of the survey with a purposive sample of researchers active in the field of trials and pilot studies, and a workshop at the 2013 Society for Clinical Trials Conference in Boston, we further refined the definitions, checklists, survey introduction and added additional questions.

**Fig 2:**
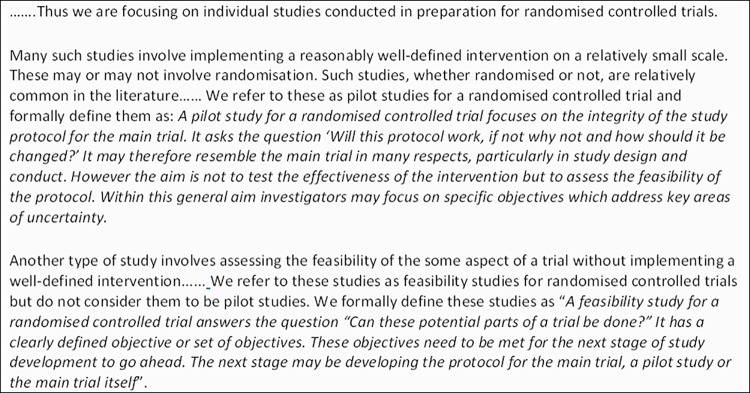
Definitions of pilot and feasibility studies used in on-line Delphi survey.

**Fig 3:**
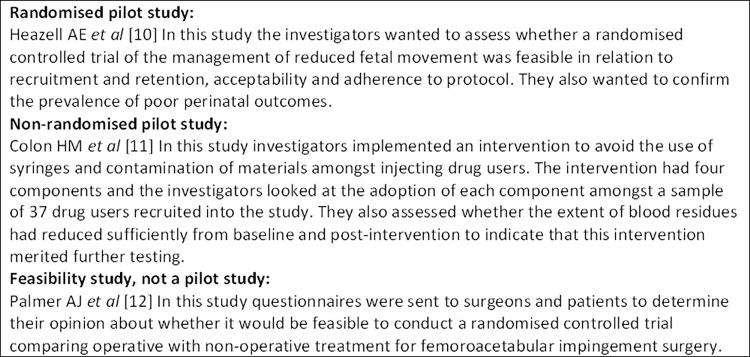
Examples of different types of pilot and feasibility study used in the on-line Delphi survey [10, 11, 12].

The first round of the main Delphi survey included: a description and explanation of our definitions of pilot and feasibility studies including examples (Figs 2 and 3); questions about participants’ characteristics; 67 proposed items for the two checklists and questions about overall appropriateness of the guidelines for feasibility or pilot studies; and four questions related to the definitions of feasibility and pilot studies: *How appropriate do you think our definition for a pilot study conducted in preparation for an RCT is*? *How appropriate do you think our definition for a feasibility study conducted in preparation for an RCT is*? *How appropriate is the way we have distinguished between two different types of study conducted in preparation for an RCT*? *How appropriate are the labels ‘pilot’ and ‘feasibility’ for the two types of study we have distinguished*? Participants were asked to rate their answers to the four questions on a nine-point scale from ‘not at all appropriate’ to ‘completely appropriate’. There was also a space for open comments about the definitions. The second round included results from the first round and again asked for further comments about the definitions.

Participants for the main survey were identified as likely users of the checklist including trialists, methodologists, statisticians, funders and journal editors. Three hundred and seventy potential participants were approached by email from the project team or directly from Clinvivo. These were individuals identified based on personal networks, authors of relevant studies in the literature, members of the Canadian Institute of Health Research, Biostatistics section of Statistics Society of Canada, and the American Statistical Society. The International Society for Clinical Biostatistics and the Society for Clinical Trials kindly forwarded our email to their entire membership. There was a link within the email to the on-line questionnaire. Each round lasted three weeks and participants were sent one reminder a week before the closure of each survey. The survey took place between August and October 2013. Ethical approval was granted by the ScHARR research ethics committee at the University of Sheffield.

### Developing a conceptual framework—Open meeting and research team meetings

The results of the Delphi survey pertaining to the definitions of feasibility and pilot studies were presented to an open meeting at the 2^nd^ UK MRC Trials Methodology Conference in Edinburgh in November 2013 [13]. Attendees chose their preferred proposition from four propositions regarding the definitions, based variously on our original definitions, the NIHR and MRC views of pilot and feasibility studies and different views expressed in the Delphi survey. At a subsequent two-day research team meeting we collated the findings from the Delphi survey and the open meeting, and considered definitions of piloting and feasiblity outside the health research context found from on-line searches using the terms ‘pilot definition’, ‘feasiblity definition’, ‘pilot study definition’ and ‘feasibility study definition’ in Google. We expected all searches to give a very large number of hits and examined the first two pages of hits only from each search. From this, we developed a conceptual framework reflecting consensus about the definitions, types and roles of feasibility and pilot studies conducted in preparation for an RCT evaluating the effect of an intervention or therapy. To ensure we incorporated the views of all researchers likely to be conducting pilot/feasiblity studies, two qualitative researchers joined the second day of the meeting which focused on agreeing this framework. Throughout this process we continually referred back to examples that we had identified to check that our emerging definitions were workable.

### Validating the conceptual framework—systematic review

To validate the proposed conceptual framework, we identified a selection of recently reported studies that fitted our definition of pilot and feasibility studies, and tested a number of hypotheses in relation to these studies. We expected that approximately 30 reports would be sufficient to test the hypotheses. We conducted a systematic review to identify studies that authors described as pilot or feasibility studies, by searching Medline via PubMed for studies that had the words ‘pilot’ or ‘feasibility’ in the title. To increase the likelihood that the studies would be those conducted in preparation for a randomised controlled trial of the effect of a therapy or intervention we limited our search to those that contained the word ‘trial’ in the title or abstract. For full details of the search strategy see S1 Fig.

To focus on current practice, we selected the 150 most recent studies from those identified by the electronic search. We did not exclude protocols since we were primarily interested in identifying the way researchers characterised their study and any possible future study and the relationship between them; we expected investigators to describe these aspects of their studies in a similar way in protocols and reports of findings. Two research team members independently reviewed study abstracts to assess whether each study fitted our working definition of a pilot or feasibility study in preparation for an RCT evaluating the effect of an intervention or therapy. Where reviewers disagreed, studies were classed as ‘possible inclusions’ and disagreements resolved by discussion with referral to the full text of the paper as necessary. Given the difficulty of interpreting some reports and to ensure that all research team members agreed on inclusion, the whole team then reviewed relevant extracted sections of the papers provisionally agreed for inclusion. We recognised that abstracts of some studies might not include appropriate information, and therefore that our initial abstract review could have excluded some relevant studies; we explored the extent of this potential omission of studies by reviewing the full texts of a random sample of 30 studies from the original 150. Since our prime goal was to identify a manageable number of relevant studies in order to test our hypotheses rather than identify all possible relevant studies we did not include any additional studies as a result of this exploratory study.

We postulated that the following hypotheses would support our conceptual framework:

1. The words ‘pilot’ and ‘feasibility’ are both used in the literature to describe studies undertaken in preparation for an RCT evaluating the effect of an intervention or therapy
2. It is possible to identify a subset of studies within the literature that are RCTs conducted in preparation for a larger RCT which evaluates the effect of an intervention or therapy. Authors do not use the term ‘pilot trial’ consistently in relation to these studies.
3. Within the literature it is not possible to apply unique mutually exclusive definitions of pilot and feasibility studies in preparation for an RCT evaluating the effect of an intervention or therapy that are consistent with the way authors describe their studies.
4. Amongst feasibility studies in preparation for an RCT which evaluates the effect of an intervention or therapy it is possible to identify some studies that are not pilot studies as defined within our conceptual framework, but are studies that acquire information about the feasibility of applying an intervention in a future study.

In order to explore these hypotheses, we categorised included studies into three groups that tallied with our framework (see results for details): randomised pilot studies, non-randomised pilot studies, feasibility studies that are not pilot studies. We also extracted data on objectives, and the phrases that indicated that the studies were conducted in preparation for a subsequent RCT.

### Validating the conceptual framework—Consensus meeting

We also took an explanation and visual representation of our framework to an international consensus meeting primarily designed to reach consensus on an extension of the 2010 CONSORT statement to randomised pilot studies. There were 19 invited participants with known expertise, experience, or interest in pilot and feasibility studies, including representatives of CONSORT, funders, journal editors, and those who had been involved in writing the NIHR definitions of pilot and feasibility studies and the MRC guidance on designing and evaluating complex interventions. Thus this was an ideal forum in which to discuss the framework also. This project was not concerned with any specific disease, and was methodological in design; no patients or public were involved.

## Results

### Developing a conceptual framework—Delphi study

**Fig 4:**
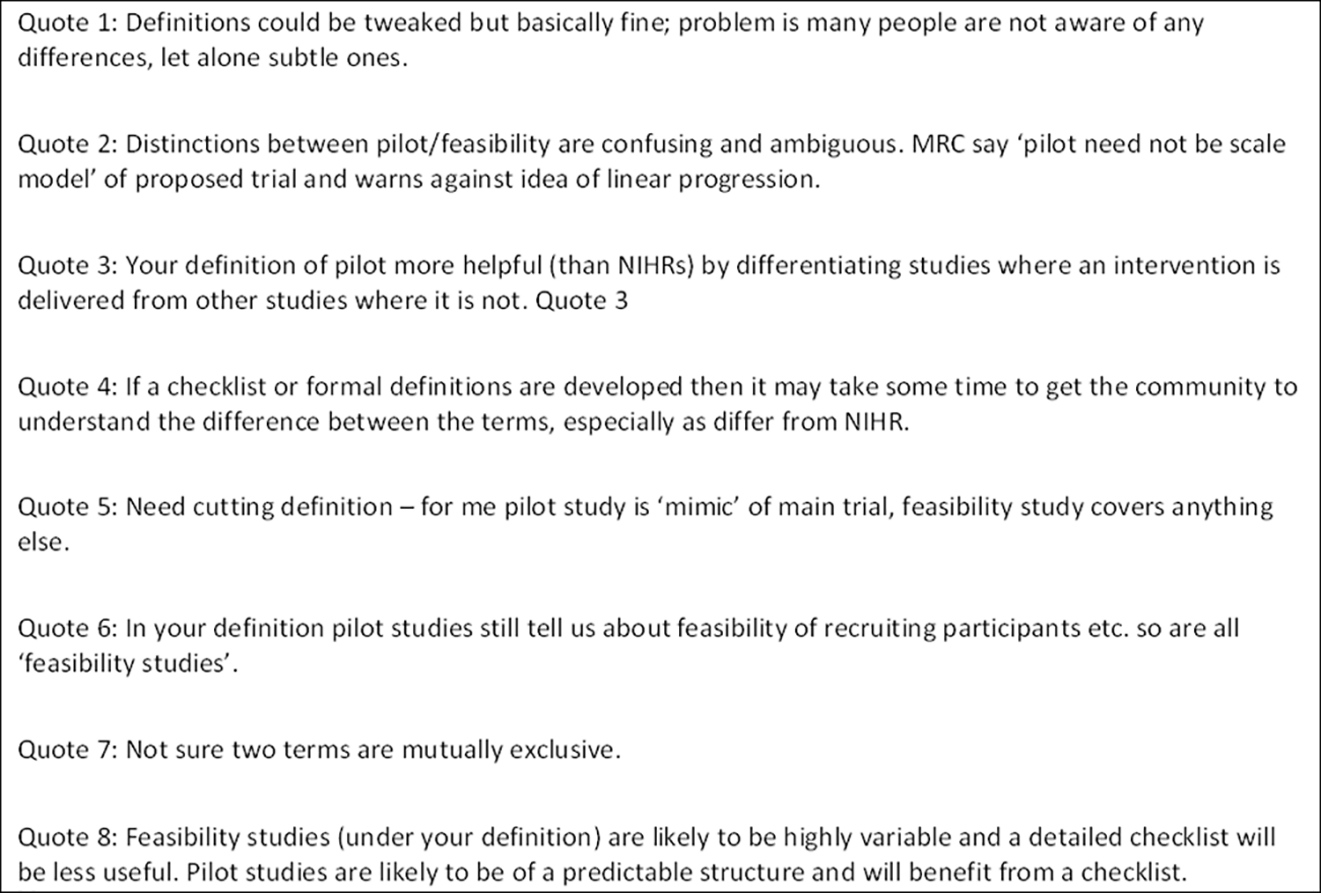
Quotes from the on-line Delphi survey.

Ninety-three individuals, including chief investigators, statisticians, trial managers, clinicians, research assistants and a funder, participated in the first round of the Delphi survey and 79 in the second round. Over 70% of participants in the first round felt that our definitions, the way we had distinguished between pilot and feasibility studies, and the labels ‘pilot’ and ‘feasibility’ were appropriate. However, these four items had some of the lowest appropriateness ratings in the survey and there were a large number of comments both in direct response to our four survey items related to appropriateness of definitions, and in open comment boxes elsewhere in the survey. Some of these comments are presented in Fig 4. Some participants commented favourably on the definitions we had drawn up (quote 1) but others were confused by them (quote 2). Several compared our definitions to the NIHR definitions pointing out the differences (quote 3) and suggesting this might make it particularly difficult for the research community to understand our definitions (quote 4). Some expressed their own views about the definitions (quote 5); largely these tallied with the NIHR definitions. Others noted that both the concept of feasibility and the word itself were often used in relation to studies which investigators referred to as pilot studies (quote 6). Others questioned whether it was practically and/or theoretically possible to make a distinction between pilot and feasibility studies (quote 6, quote 7), suggesting that the two terms are not mutually exclusive and that feasibility was more of an umbrella term for studies conducted prior to the main trial. Some participants felt that, using our definitions, feasibility studies would be less structured and more variable and therefore their quality would be less appropriately assessed via a checklist (quote 8). These responses regarding definitions mirrored what we had found in the user-testing of the Delphi survey, the Society for Clinical Trials workshop, and differences of opinion already apparent in the literature. In the second round of the survey there were few comments about definitions.

### Developing a conceptual framework—Open meeting and research team meetings

**Fig 5:**
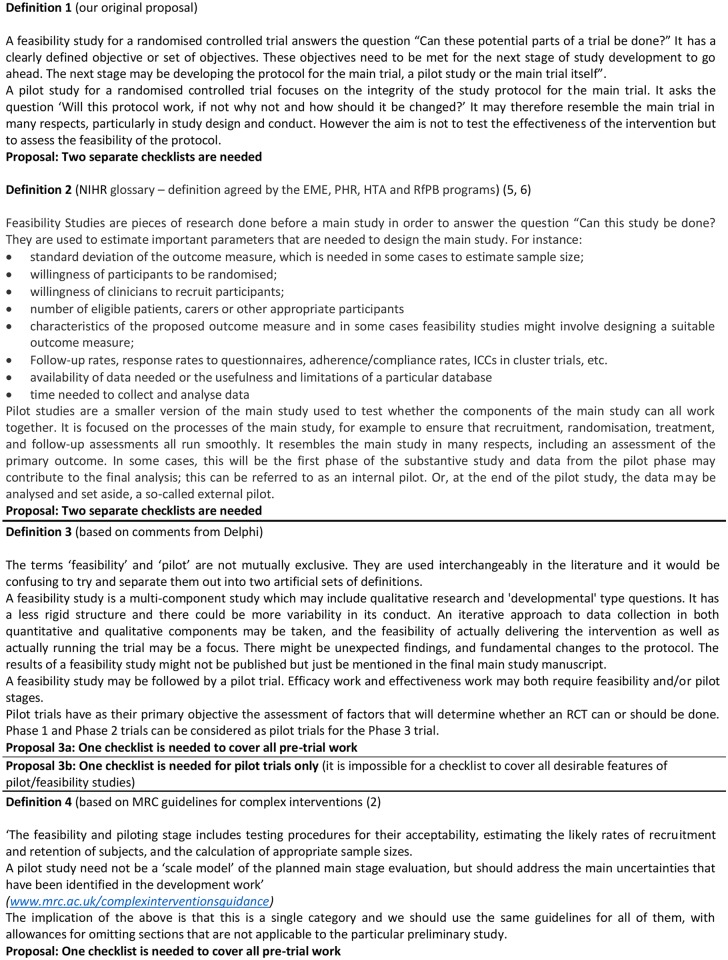
Four propositions presented at Edinburgh open meeting.

There was a wide range of participants in the open meeting, including senior quantitative and qualitative methodologists, and a funding body representative. The four propositions we devised to cover different views about definitions of pilot and feasibility studies are shown in Fig 5. Fourteen out of the fifteen attendees who voted on these propositions preferred propositions 3 or 4, based on comments from the Delphi survey and the MRC guidance on designing and evaluating complex interventions respectively. Neither of these propositions implied mutually exclusive definitions of pilot and feasibility studies.

Definitions of feasibility outside the health research context focus on the likelihood of being able to do something. For example, the Oxford on-line dictionary defines feasibility as: ‘The state or degree of being easily or conveniently done’ [14] and a feasibility study as: ‘An assessment of the practicality of a proposed plan or method’ [15]. Some definitions also suggest that a feasibility study should help with decision making, for example [16]: ‘The feasibility study is an evaluation and analysis of the potential of a proposed project. It is based on extensive investigation and research to support the process of decision making’. Outside the health research context the word ‘pilot’ has several different meanings but definitions of pilot studies usually focus on an experiment, project or development undertaken in advance of a future wider experiment, project or development. For example the Oxford on-line dictionary describes a pilot study as: ‘Done as an experiment or test before being introduced more widely’ [17]. Several definitions carry with them ideas that the purpose of a pilot study is also to facilitate decision making, for example ‘a small-scale experiment or set of observations undertaken to decide how and whether to launch a full-scale project’ [18] and some definitions specifically mention feasibility, for example: ‘a small scale preliminary study conducted in order to evaluate feasibility’ [19].

In keeping with these definitions not directly related to the health research context, we agreed that feasiblity is a concept encapsulating ideas about whether it is possible to do something and that *a feasibility study asks whether something can be done*, *should we proceed with it*, *and if so*, *how*. While piloting is also concerned with whether something can be done and whether and how we should proceed with it, it has a further dimension; piloting is implementing something, or part of something, in a way you intend to do it in future to see whether it can be done in practice. We therefore agreed that *a pilot study is a study in which a future study or part of a future study*, *is conducted on a smaller scale to ask the question whether something can be done*, *should we proceed with it*, *and if so*, *how*. The corollary of these definitions is that all pilot studies are feasibility studies but not all feasibility studies are pilot studies. Within the context of RCTs, the focus of our research, the ‘something’ in the definitions can be replaced with ‘a future RCT evaluating the effect of an intervention or therapy’. Studies that address the question of whether the RCT can be done, should we proceed with it and if so how, can then be classed as feasibility or pilot studies. Some of these studies may, of course, have other objectives but if they are mainly focusing on feasiblity of the future RCT we would include them as feasiblity studies. All three studies used as examples in our Delphi survey [10–12] satisfy the definition of a feasiblity study. However, a study by Piot *et al*, that we encountered while developing the Delphi study, does not. This study is described as a pilot trial in the abstract but the authors present only data on effectiveness and although they state that their results require confirmation in a larger study it is not clear that their pilot study was conducted in preparation for such a larger study [20]. On the other hand, Palmer *et al* ‘performed a feasibility study to determine whether patient and surgeon opinion was permissive for a Randomised Controlled Trial (RCT) comparing operative with non-operative treatment for FAI [femoroacetabular impingement]’ [12]. Heazell *et al* describe the aim of their randomised study as ‘to address whether a randomised controlled trial (RCT) of the management of RFM [reduced fetal movement] was feasible’ [10]. Their study was piloting many of the aspects they hoped to implement in a larger trial of RFM, thus making this also a pilot study, whereas the study conducted by Palmer *et al*, which comprised a questionnare to clinicians and seeking patient opinion, is not a pilot study but is a feasibility study.

Within our framework, some important studies conducted in advance of a future RCT to evaluate the effect of a therapy or intervention are not feasibility studies. For example, a systematic review, usually an essential pre-requisite for such an RCT, normally addresses whether the future RCT is *necessary* or *desirable*, not whether it is *feasible*. To reflect this, we developed a comprehensive diagrammatical representation of our framework for studies conducted in preparation for an RCT which, for completeness, includes, on the left hand side, early studies that are not pilot and feasibility studies, such as systematic reviews and, along the bottom, details of existing or planned reporting guidelines for different types of study (S2 Fig).

### Validating the conceptual framework—Systematic review

**Fig 6:**
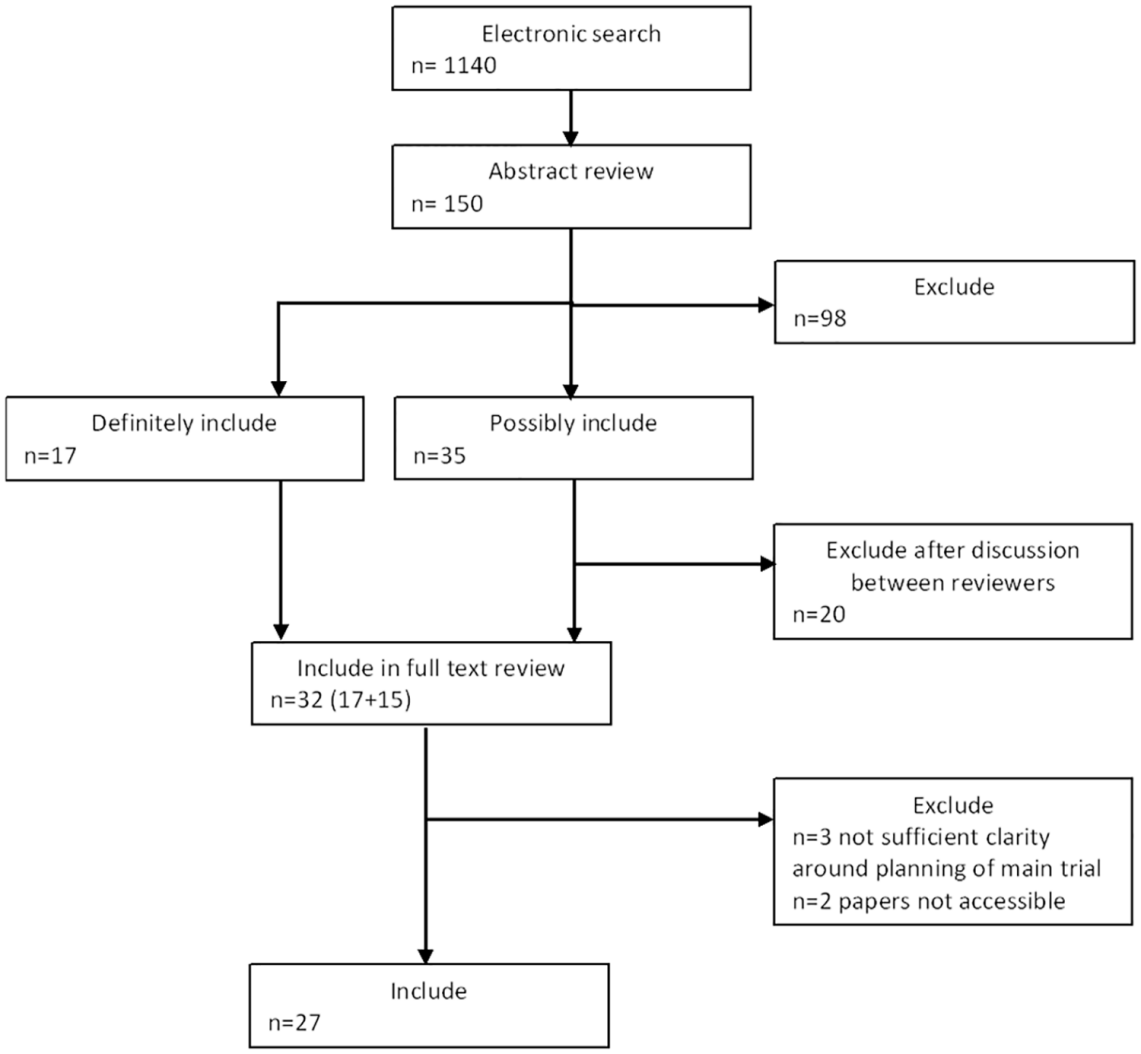
Flow chart showing identification of empirical pilot and feasibility studies.

From the 150 most recent studies identified by our electronic search, we identified 27 eligible reports (Fig 6). In keeping with our working definition of a pilot or feasibility study, to be included the reports had to show evidence that investigators were addressing at least some feasibility objectives and that the study was in preparation for a future RCT evaluating the effect of an intervention. Ideally we would have stipulated that the primary objective of the study should be a feasibility objective but, given the nature of the reporting of most of these studies, we felt this would be too restrictive.

The 27 studies are reported in Table 1 and results relating to terminology that authors used summarised in Table 2. Results in Table 2 support our first hypothesis that the words ‘pilot’ and ‘feasibility’ are both used in the literature to describe studies undertaken in preparation for a randomised controlled trial of effectiveness; 63% (17/27) used both terms somewhere in the title or abstract. The table also supports our second hypothesis that amongst the subset of feasibility studies in preparation for an RCT that are themselves RCTs, authors do not use the term ‘pilot trial’ consistently in relation to these studies; of the 18 randomised studies only eight contained the words ‘pilot’ and ‘trial’ in the title. Our third hypothesis, namely that it is not possible to apply unique mutually exclusive definitions of pilot and feasibility studies in preparation for an RCT that are consistent with the way authors describe their studies, is supported by the characteristics of studies presented in Table 1 and summarised in Table 2. We could find no design or other features (such as randomisation or presence of a control group) that distinguished between those that investigators called feasibility studies and those that they called pilot studies. However, the fourth hypothesis, that amongst studies in preparation for an RCT evaluating the effect of an intervention or therapy it is possible to identify some studies that explore the feasibility of a certain intervention or acquire related information about the feasibility of applying an intervention in a future study but are not pilot studies, was not supported; we identified no such studies amongst those reported in Table 1. Nevertheless, we had identified two prior to carrying out the review [10, 15].

**Table 1:** Categorisation and description of the 27 pilot/feasibility studies identified in our systematic review.

	Pilot in title or abstract	Feasibility in title or abstract	Objectives	Phrase indicating that this is a pilot/feasibility study in preparation for a future definitive trial	Trial in title or abstract
*Randomised pilot/feasibility studies*					
Allen [21]	-	Title and abstract	The study purpose was to assess the feasibility of recruiting pregnant adolescents into a randomised controlled trial, in order to inform the design of an adequately powered trial which could test the effect of caseload midwifery on preterm birth for pregnant adolescents.	…. in order to inform the design of an adequately powered trial which could test the effect of caseload midwifery on preterm birth for pregnant adolescents.	Title and abstract
Boogerd [22]	-	Title and abstract	To evaluate the feasibility of an online interactive treatment environment for adolescents with type 1 diabetes, called Sugarsquare, to supplement usual care	Results are promising and next steps are a full-scale randomised controlled trial and subsequent implementation in daily care.	Abstract
Buse [23]	Title and abstract	Abstract	We undertook a pilot trial to determine the feasibility of a trial comparing accelerated care (i.e., rapid medical clearance and surgery) and standard care among patients with a hip fracture.	These results show the feasibility of a trial comparing accelerated and standard care among patients with hip fracture and support a definitive trial….. Finally, this pilot trial identified design issues that we were able to overcome through protocol amendments.	Title and abstract
Clark [24]	Title and abstract	Abstract	The primary aim of this pilot trial was to assess the feasibility and safety of asking adults with stage 3 CKD to follow the above hydration intervention.	Prior to initiating a larger randomised controlled trial (RCT), we examined the safety and feasibility of asking adults with chronic kidney disease (CKD) to increase their water intake.	Title and abstract
Crawley [25]	Abstract	Title and abstract	Integrated qualitative methodology was used to explore the feasibility and acceptability of the recruitment, randomisation and interventions.	As the aim of this study was to assess the feasibility of a future definitive trial, we did not undertake a formal sample size calculation.	Title and abstract
Goodall [26]	Title and abstract	-	To this end, our trial had three objectives: piloting of trial processes; a quantitative measurement of changes in heart healthy behaviours with an economic evaluation (results published) and a qualitative evaluation of LHTs training and intervention delivery, implementation and acceptability (results to be reported elsewhere).	Our pilot explored feasibility of an LHT intervention before embarking on a full RCT.	Title and abstract
Higgins [27]	Title	Abstract	Evaluate the feasibility of a randomized controlled trial aimed at determining the efficacy of rTMS as an adjunct to task-oriented therapy in facilitating restoration of arm function after stroke.	Evaluate the feasibility of a randomized controlled trial….	Title and abstract
Holt [28]	Title and abstract	Abstract	We plan a large, definitive, primary-care-based trial to determine efficacy and safety in patients with rotator cuff tendinopathy, and conducted a pilot trial to explore feasibility.	The lessons learned from this pilot will usefully inform the design of a large, definitive efficacy trial in primary care.	Title and abstract
Hurt [29]	Abstract	Title and abstract	This trial will assess the feasibility and inform the design of a large, UK-wide, clinical trial of a change to the NICE guidelines for urgent referral for chest X-ray for suspected lung cancer.	…..and inform the design of a large, UK-wide, clinical trial….	Title and abstract
Lakes [30]	Title and abstract	Title	The objective of this pilot study was to evaluate Taekwondo implemented in public middle school physical education (PE)…..Together, academic and community partners developed the current pilot study to address the feasibility and acceptability of implementing Taekwondo into PE in a public, low-income middle school as well as to investigate the effects of Taekwondo	Therefore, this pilot study lacked sufficient power to measure effects with statistical significance, but was expected to be sufficient to note trends in improvements that could be studied in a subsequent larger study.	Abstract
Lee [31]	Title and abstract	Abstract	Here, we examine the feasibility of the BCI system with a new game that incorporates memory training in improving memory and attention in a pilot sample of healthy elderly.	Obtain an estimate of efficacy in improving memory and attention in healthy elderly participants to determine whether the study should proceed to a phase III trial.	Abstract
McKenna [32]	-	Title and abstract	The aim of this randomized controlled trial was to evaluate the feasibility of delivering the Bridges stroke self-management program in addition to usual stroke rehabilitation compared with usual rehabilitation only.	A range of outcome measures were used to test their feasibility and explore whether they would be meaningful to use in a fully powered trial.……it would be advisable in future trials to keep more detailed records regarding the time spent on each component.	Title and abstract
Powell [33]	Title and abstract	Title and abstract	This article presents the findings of a pilot economic evaluation study running alongside the Bristol Girls Dance Project (BGDP) feasibility study.	…using a pilot economic evaluation to inform design of a full trial	Title and abstract
Saez [34]	Title and abstract	Abstract	In this work, we present the results of a randomized pilot study to evaluate the feasibility and to define the potential value for clinical practice of Curiam BT,…	We used these results as a baseline for the estimation of the total number of cases required to obtain statistical significant difference (α = .05) in a larger RCT for the discrimination of tumour grades (Q2).	Abstract
Safdar [35]	Title and abstract	-	We aim to develop and evaluate a behavioural intervention ‘Smoke Free Homes’ (SFH) for TB patients that encourages them to negotiate a smoke free environment within their homes.	This is a pilot individual randomised controlled trial of SFH that will inform the design of a future definitive trial.	Title and abstract
Schoultz [36]	Title	Abstract	The aim of this study is to obtain the information required to design a full scale randomised controlled trial (RCT) that will examine the effectiveness of MBCT in improving quality of life for IBD patients.	The data will inform the estimate for recruitment rates for a full trial	Title and abstract
Siriwardhana [37]	Title and abstract	Abstract	The proposed pilot study aims to explore the feasibility of integrating mental health care into primary care by providing training to primary care practitioners serving displaced populations, in order to improve identification, treatment,and referral of patients with common mental disorders via the World Health Organization Mental Health Gap Action	Results will be used to formulate sample size calculation for a larger intervention.	Abstract
Wolf [38]	Title and abstract	-	The aim of the work presented here is to reduce the number of falls on a geriatric ward by monitoring patients more closely. To achieve this goal, a bed-exit alarm that reliably detects an attempt to get up has been constructed.	There are plans for a larger multicenter clinical trial to fortify these results. However, to be able to equip clinics on a larger scale and reach more patients, some modifications to the hardware are needed.	Abstract
*Non-randomised pilot/feasibility studies*					
Alers [39]	Title	-	A phase I clinical trial to investigate the efficacy of maternal oral melatonin administration in women with a pregnancy complicated by fetal growth restriction	If this trial is successful, the results will be used to inform future randomised controlled trials.	Title and abstract
Carlesso [40]	Title and abstract	Title and abstract	To pilot and determine the feasibility of estimating adverse events in patients with neck pain treated with cervical manipulation/mobilization by Canadian orthopaedic manual physiotherapists (OMPTs) using an online data-collection system to provide estimates…..	…..to provide estimates for a future larger multi-centre international study.	Abstract
Collado [41]	Title	-	to evaluate BATD, an idiographic intervention, employing the rationale that BATD provides a flexible and easily-tailored treatment framework able to address the individual and psychological needs of depressed Latinos.	The study’s positive outcomes suggest that a Stage II randomized clinical trial is a logical next step.	Abstract
Galantino [42]	Abstract	Title and abstract	This study aimed to determine the feasibility of tai chi to improve well-being for women experiencing AI-associated arthralgias (AIAAs).	The sample size of this pilot study was not intended to provide an efficacy analysis but rather to obtain an estimate of the effect size and variance necessary to plan a definitive study to test and refine individual components of the tai chi protocol for AIAA and measurement tools.	Abstract
Garcia [43]	Title and abstract	Abstract	Prior to implementing a large randomized trial at our institution, we investigated the feasibility, safety, and initial efficacy of acupuncture for uncontrolled pain among cancer patients.	Prior to implementing a large randomized trial at our institution……	Abstract
Hu [44]	-	Title and abstract	To determine the feasibility of all aspects of a pragmatic observational study designed: (1) to evaluate the effectiveness and cost effectiveness of integrated treatments for MSDs in an integrated NHS hospital in the UK; (2) to determine the acceptability of the study design and research process to patients; (3) to explore patients' expectation and experience of receiving integrated treatments.	It will inform the design of a future trial including recruitment, retention, suitability of the outcome measures and patients’ experiences.	Abstract
Misumi [45]	-	Title and abstract	We conducted a feasibility study to evaluate the safety and efficacy of carboplatin plus irinotecan in preparation for a planned Phase III study.	Based on these results, a Phase II/III trial comparing carboplatin plus etoposide with carboplatin plus irinotecan for elderly patients with extensive disease small-cell lung cancer is being planned by the Japan Clinical Oncology Group.	Abstract
Penn [46]	Title and abstract	Title and abstract	…aimed to assess the feasibility, acceptability and outcomes at a 12-month follow-up of a behavioural intervention for adults at risk of T2D.	Feasibility and acceptability of this novel intervention were assessed in preparation for a definitive effectiveness trial.	Abstract
Pompeu [47]	Title	Title and abstract	To assess the feasibility, safety and outcomes of playing Microsoft Kinect AdventuresTM for people with Parkinson’s disease in order to guide the design of a randomised clinical trial.	…. in order to guide the design of a randomised clinical trial.	Abstract

**Table 2:** Summary of terms used in 27 pilot/feasibility studies.

Use of the terms pilot and feasibility in the title and abstract	All included studies	Randomised studies	Non-randomised studies	Randomised studies with trial in the title
Pilot in title, no mention of feasibility in title or abstract	5	3	2	2
Feasibility in title, no mention of pilot in title or abstract	5	3	2	2
Both terms in title	5	2	3	1
Pilot in title, feasibility in abstract only	9	8	1	5
Feasibility in title, pilot in abstract only	3	2	1	2
Total	27	18	9	12

Out of our exploratory sample of 30 study reports for which we reviewed full texts rather than only titles and abstracts, we identified 10 that could be classed as pilot or feasibility studies using our framework. We had already identified four of these in our sample reported in Table 1, but had failed to identify the other six. As expected, this was because key information to identify them as pilot or feasiblity studies such as the fact that they were in preparation for a larger RCT, or that the main objectives were to do with feasiblity were not included in the abstract. Thus our assumption that an initial screen using only abstracts resulted in the omission of some pilot and feasiblity studies was correct.

### Validating the conceptual framework—Consensus meeting

**Fig 7:**
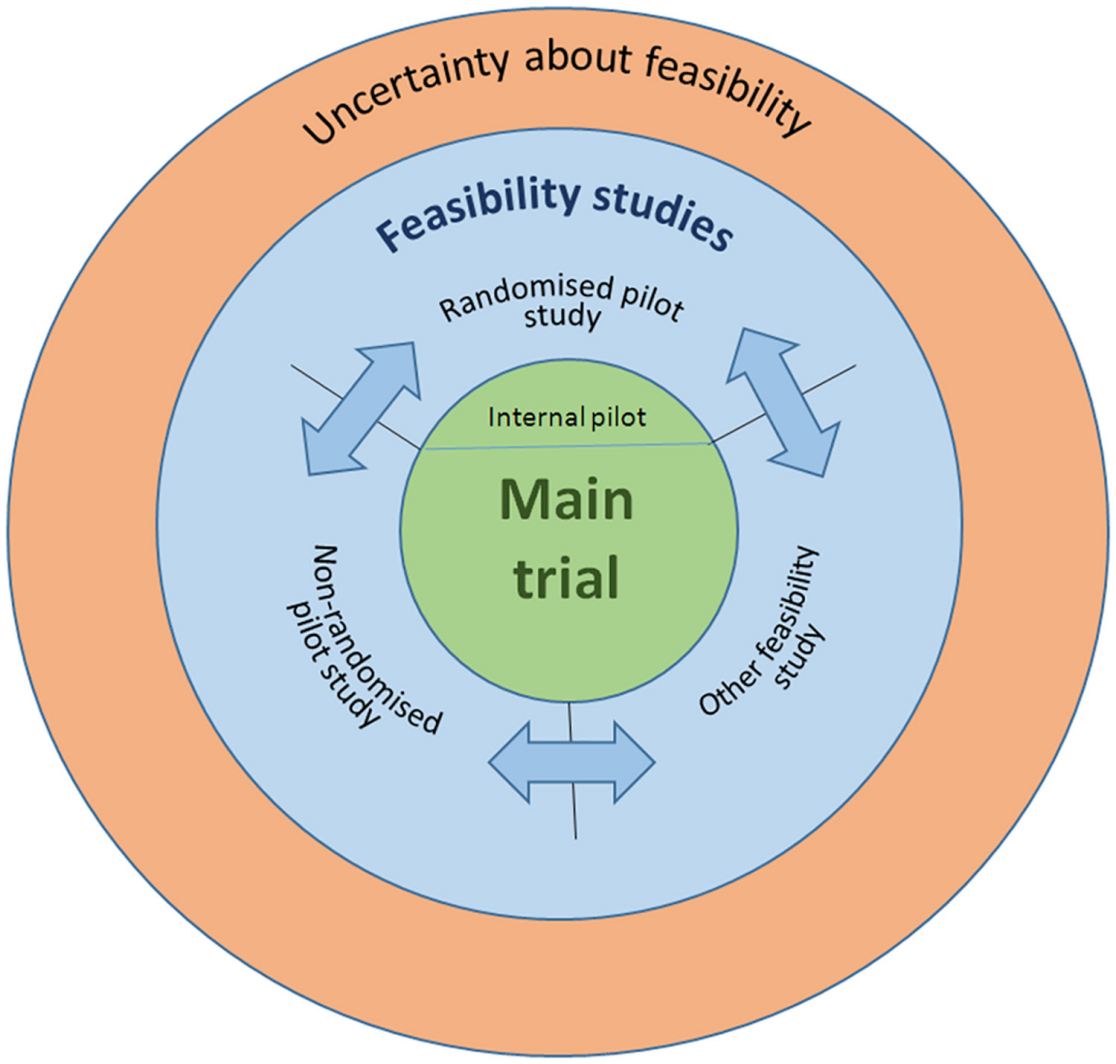
Conceptual framework.

International consensus meeting participants agreed with the general tenets of our conceptual framework including the ideas that all pilot studies are feasibility studies but that some feasibility studies are not pilot studies. They suggested that any definitive diagrammatic representation should more strongly reflect non-linearity in the ordering of feasibility studies. As a result of their input we produced a new, simplified, diagrammatical representation of the framework (Fig 7) which focuses on the key elements represented inside an oval shape on our original diagram, omits the wider context outside this shape, and highlights some features, including the non-linearity, more clearly.

### The finalised framework

Fig 7 represents the framework. The figure indicates that where there is uncertainty about future RCT feasibility, a feasibility study is appropriate. Feasibility is thus an overarching concept within which we distinguish between three distinct types of study. *Randomised pilot studies* are those studies in which the future RCT, or parts of it, including the randomisation of participants, is conducted on a smaller scale (piloted) to see if it can be done. Thus randomised pilot studies can include studies that for the most part reflect the design of a future definitive trial but, if necessary due to remaining uncertainty, may involve trying out alternative strategies, for example, collecting an outcome variable via telephone for some participants and on-line for others. Within the framework randomised pilot studies could also legitimately be called randomised feasibility studies. Two-thirds of the studies presented in Table 1 are of this type.

*Non-randomised pilot studies* are similar to randomised pilot studies; they are studies in which all or part of the intervention to be evaluated and other processes to be undertaken in a future trial is/are carried out (piloted) but without randomisation of participants. These could also legitimately be called by the umbrella term, feasibility study. These studies cover a wide range from those that are very similar to randomised pilot studies except that the intervention and control groups have not been randomised, to those in which only the intervention, and no other trial processes, are piloted. One-third of studies presented in Table 1 are of this type.

*Feasibility studies that are not pilot studies* are those in which investigators attempt to answer a question about whether some element of the future trial can be done but do not implement the intervention to be evaluated or other processes to be undertaken in a future trial, though they may be addressing intervention development in some way. Such studies are rarer than the other types of feasibility study and, in fact, none of the studies in Table 1 were of this type. Nevertheless, we include these studies within the framework because they do exist; the Palmer study [15] in which surgeons and patients were asked about the feasibility of randomisation is one such example. Other examples might be interviews to ascertain the acceptability of an intervention, or questionnaires to assess the types of outcomes participants might think important. Within the framework these studies can be called feasibility studies but cannot be called pilot studies since no part of the future randomised controlled trial is being conducted on a smaller scale.

Investigators may conduct a number of studies to assess feasibility of an RCT to test the effect of any intervention or therapy. While it may be most common to carry out what we have referred to as *feasibility studies that are not pilot studies* before *non-randomised pilot studies*, and *non-randomised pilot studies* prior to *randomised pilot studies*, the process of feasibility work is not necessarily linear and such studies can in fact be conducted in any order. For completeness the diagram indicates the location of internal pilot studies.

## Discussion

There are diverse views about the definitions of pilot and feasibility studies within the research community. We reached consensus over a conceptual framework for the definitions of these studies in which feasibility is an overarching concept for studies assessing whether a future study, project or development can be done. For studies conducted in preparation for a RCT assessing the effect of a therapy or intervention, three distinct types of study come under the umbrella of feasibility studies: randomised pilot studies, non-randomised pilot studies, feasibility studies that are not pilot studies. Thus pilot studies are a subset of feasibility studies. A review of the literature confirmed that it is not possible to apply mutually exclusive definitions of pilot and feasibility studies in preparation for such an RCT that are consistent with the way authors describe their studies. For example Lee *et al* [31], Boogerd *et al* [22] and Wolf *et al* [38] all describe randomised studies exploring the feasibility of introducing new systems (brain computer interface memory training game, on-line interactive treatment environment, bed-exit alarm respectively) but Lee *et al* describe their study as a ‘A Randomized Control Pilot Study’, with the word ‘feasibility’ used in the abstract and text, while the study by Boogerd *et al*. is titled ‘Teaming up: feasibility of an online treatment environment for adolescents with type 1 diabetes’, and Wolf at al describe their study as a pilot study without using the word ‘feasibility’.

Our re-evaluation of the definitions of pilot and feasibility studies was conducted over a period of time with input via a variety of media by multi-disciplinary and international researchers, publishers, editors and funders. It was to some extent a by-product of our work developing reporting guidelines for such studies. Nevertheless, we were able to gather a wide range of expert views, and the iterative nature of the development of our thinking has been an important part of obtaining consensus. Other parallel developments, including the recent establishment of the new Pilot and Feasibility Studies journal [48], suggest that our work is, indeed, timely. We encountered several difficulties in reviewing empirical study reports. Firstly, it was sometimes hard to assess whether studies were planned in preparation for an RCT or whether the authors were conducting a small study and simply commenting on the fact that a larger RCT would be useful. Secondly, objectives were sometimes unclear, and/or effectiveness objectives were often emphasised in spite of recommendations that pilot and feasibility studies should not be focusing on effectiveness [1, 4]. In identifying relevant studies we erred on the side of inclusiveness, acknowledging that getting these studies published is not easy and that there are, as yet, no definitive reporting guidelines for investigators to follow. Lastly, our electronic search was unable to identify any feasibility studies that were not pilot studies according to our definitions. Subsequent discussion with qualitative researchers suggested that this is because such studies are often not described as feasibility studies in the title or abstract.

Our framework is compatible with the UK MRC guidance on complex interventions which suggests a ‘feasibility and piloting’ phase as part of the work to design and evaluate such interventions without any explicit distinction between pilot and feasibility studies. In addition, although our framework has a different underlying principle from that adopted by UK NIHR, the NIHR definition of a pilot study is not far from the subset of studies we have described as randomised pilot studies. Although there appears to be increasing interest in pilot and feasibility studies, as far as we are aware no other funding bodies specifically address the nature of such studies. The National Institute for Health in the USA does, however, routinely require published pilot studies before considering funding applications for certain streams, and the Canadian Institutes of Health Research routinely have calls for pilot or feasibility studies in different clinical areas to gather evidence necessary to determine the viability of new research directions determined by their strategic funding plans. These approaches highlight the need for clarity regarding what constitutes a pilot study.

There are several previous reviews of empirical pilot and feasibility studies [1, 4, 7]. In the most recent, reviewing studies published between 2000 and 2009 [7], the authors identified a large number of studies, described similar difficulty in identifying whether a larger study was actually being planned, and similar lack of consistency in the way the terms ‘pilot’ and ‘feasibility’ are used. Nevertheless, in methodological work, many researchers have adopted fairly rigid definitions of pilot and feasibility studies. For example, Bugge *et al* in developing the ADEPT framework refer to the NIHR definitions and suggest that feasibility studies ask questions about ‘whether the study can be done’ while pilot trials are ‘(a miniature version of the main trial), which aim to test aspects of study design and processes for the implementation of a larger main trial in the future’ [49]. Although not explicitly stated, the text seems to suggest that pilot and feasibility studies are mutually exclusive. Our work indicates that this is neither necessary nor desirable. There is, however, general agreement in the literature about the purpose of pilot and feasibility studies. For example, pilot trials are ‘to provide sufficient assurance to enable a larger definitive trial to be undertaken’ [50], and pilot studies are ‘designed to test the performance characteristics and capabilities of study designs, measures, procedures, recruitment criteria, and operational strategies that are under consideration for use in a subsequent, often larger, study’ [51], and ‘play a pivotal role in the planning of large-scale and often expensive investigations’ [52]. Within our framework we define all studies aiming to assess whether a future RCT is do-able as ‘feasibility studies’. Some might argue that the focus of their study in preparation for a future RCT is acceptability rather than feasibility, and indeed, in other frameworks, such as the RE-AIM framework [53], feasibility and acceptability are seen as two different concepts. However, it is perfectly possible to explore the acceptability of an intervention, of a data collection process or of randomisation in order to determine the *feasibility* of a putative larger RCT. Thus the use of the term ‘feasibility study’ for a study in preparation for a future RCT is not incompatible with the exploration of issues other than feasibility within the study itself.

There are numerous previous studies in which the investigators review the literature and seek the counsel of experts to develop definitions and clarify terminology. Most of these relate to clinical or physiological definitions [54–56]. A few explorations of definitions relate to concepts such as quality of life [57]. Implicit in much of this work is that from time to time definitions need rethinking as knowledge and practice moves on. From an etymological point of view this makes sense. In fact, the use of the word ‘pilot’ to mean something that is a prototype of something else only appears to emerge in the middle of the twentieth century and the first use of the word in relation to research design that we could find was in 1947—a pilot survey [58]. Thus we do not have to look very far back to see changes in the use of one of the words we have been dealing with in developing our conceptual framework. We hope what we are proposing here is helpful in the early twenty-first century to clarify the use of the words ‘pilot’ and ‘feasibility’ in a health research context.

We suggest that researchers view feasibility as an overarching concept, with all studies done in preparation for a main study open to being called feasibility studies, and with pilot studies as a subset of feasibility studies. All such studies should be labelled ‘pilot’ and/or ‘feasibility’ as appropriate, preferably in the title of a report, but if not certainly in the abstract. This recommendation applies to all studies that contribute to an assessment of the feasibility of an RCT evaluating the effect of an intervention. Using either of the terms in the title will be most helpful for those conducting future electronic searches. However, we recognise that for qualitative studies, authors may find it convenient to use the terms in the abstract rather than the title. Authors also need to describe objectives and methods well, reporting clearly if their study is in preparation for a future RCT to evaluate the effect of an intervention or therapy.

Though the focus of this work was on the definitions of pilot and feasibility studies and extensive recommendations for the conduct of these studies is outside its scope, we suggest that in choosing what type of feasibility study to conduct investigators should pay close attention to the major uncertainties that exist in relation to trial or intervention. A randomised pilot study may not be necessary to address these; in some cases it may not even be necessary to implement an intervention at all. Similarly, funders should look for a justification for the type of feasibility study that investigators propose. We have has also highlighted the need for better reporting of these studies. The CONSORT extension for randomised pilot studies that our group has developed are important in helping to address this need and will be reported separately. Nevertheless, further work will be necessary to extend or adapt these reporting guidelines for use for non-randomised pilot studies and for feasibility studies that are not pilot studies. There is also more work to be done in developing good practice guidance for the conduct of pilot and feasibility studies.

## Supporting Information

S1 Fig Search strategy to identify studies that authors described as pilot or feasibility studies. (DOCX)

S2 Fig Initial comprehensive diagrammatic representation of framework. (DOCX)
