# Supplementary material for: Defining Feasibility and Pilot Studies in Preparation for Randomised Controlled Trials: Development of a Conceptual Framework
Source: PLoS One. 2016 Mar 15;11(3):e0150205. doi: 10.1371/journal.pone.0150205 (PMC4792418; doi:10.1371/journal.pone.0150205)
Supplement: S1 Fig — (DOCX) [file pone.0150205.s001.docx]

**S1 figure: Search strategy to identify studies that authors described as pilot or feasibility studies**

| #1     pilot[Title]  #2     feasibility[Title]  #3     #1 OR #2  #4     trial[Title/Abstract]  #5     2013:2013[pDat]  #6     #3 AND #4 AND #5 |
| --- |
