# Supplementary material for: Defining Feasibility and Pilot Studies in Preparation for Randomised Controlled Trials: Development of a Conceptual Framework
Source: PLoS One. 2016 Mar 15;11(3):e0150205. doi: 10.1371/journal.pone.0150205 (PMC4792418; doi:10.1371/journal.pone.0150205)
Supplement: S2 Fig — (DOCX) [file pone.0150205.s002.docx]

**Figure 7: Initial comprehensive diagrammatic representation of framework**
